# Variation in Nanocrystalline Phase Content on Mechanical Properties and Wear Resistance of FeCrMoWBRE Amorphous/Nanocrystalline Coating Deposited by High-Velocity Arc Spraying

**DOI:** 10.3390/nano15040305

**Published:** 2025-02-17

**Authors:** Hao Du, Wei Xin, Bo Wang, Ji’an Feng, Xingchuan Xia, Yujiang Wang, Shicheng Wei

**Affiliations:** 1 School of Ocean Engineering, Guangzhou Maritime University, Guangzhou 510725, China; duhao@gzmtu.edu.cn (H.D.); jafeng039@hotmail.com (J.F.); 2 National Key Laboratory for Remanufacturing, Beijing 100072, China; xw1390332928@126.com (W.X.); wangbobo421@163.com (B.W.); wsc33333@163.com (S.W.); 3 School of Materials Science and Engineering, Hebei University of Technology, Tianjin 300130, China; xc_xia@hebut.edu.cn

**Keywords:** high-velocity arc spraying, amorphous/nanocrystalline coating, process parameters, mechanical properties, wear resistance

## Abstract

The incorporation of a homogeneously distributed nanocrystalline phase in Fe-based amorphous coatings is widely acknowledged to enhance wear resistance across various applications. In this study, FeCrMoWBRE amorphous/nanocrystalline composite coatings were fabricated on 45# steel substrates using high-velocity arc spraying (HVAS). The coatings were produced under varying spraying voltages, currents, and distances, following the Taguchi experimental design methodology. The microstructure, mechanical properties, and wear resistance of the coatings were systematically analyzed, with a particular focus on the relationship between nanocrystalline/amorphous phase content and key performance metrics, including microhardness, adhesive strength, and wear rate. A positive correlation was observed between the nanocrystalline phase content and both mechanical properties and wear resistance. The coating with optimized nanocrystalline phase content of 21.4% exhibits the lowest wear rate of 1.39 × 10^−7^ mm^3^·N^−1^·m^−1^ under a 100 N load and oil lubrication. These findings underscore the critical role of controlling the nanocrystalline phase content in Fe-based amorphous/nanocrystalline composite coatings to maximize wear resistance under oil-lubricated conditions.

## 1. Introduction

Over the past thirty years, amorphous alloys have attracted increasing attention due to their excellent physical and mechanical properties, especially when compared to traditional metallic materials [1]. As a class of non-crystalline metals or metastable solids lacking long-range atomic order, amorphous metals possess a homogeneous structure without grain boundaries, which contributes to their superior properties, including enhanced strength, hardness, and corrosion resistance [2,3,4]. In this kind of materials, Fe-based amorphous alloys are particularly appealing due to their high crystallization temperature [5], exceptional corrosion and wear resistance, high thermal stability, and relatively low material cost [6,7]. However, the poor plasticity and brittleness of amorphous alloys have limited their applications. This challenge was addressed with the development of amorphous coatings, which not only retain the desirable properties of the corresponding amorphous alloys but also overcome their inherent shortcomings.

Thermal spraying is a widely applied coating and remanufacturing technology for corrosion and wear protection in industrial applications [8,9,10]. In this process, finely divided metallic or non-metallic materials are heated to a molten or semi-molten state using a suitable heat source, such as an arc, flame, or plasma [11]. High-pressure gas is then employed to accelerate the molten droplets onto the substrate surface, forming a dense protective coating. Wear-resistant coatings for engine components are generally produced using methods such as plasma spraying [12,13], supersonic flame spraying [14,15], detonation spraying [16,17,18], and high-velocity arc spraying [19,20]. Among these techniques, high-velocity arc spraying is particularly advantageous for amorphous coatings due to its high thermal efficiency, minimal thermal impact on the workpieces, and flexibility in the working environment [21].

In fact, the Fe-based amorphous coatings produced by arc spraying typically exhibit a microstructure composed of both amorphous and nanocrystalline phases, along with the characteristic properties of thermally sprayed coatings. Traditionally, Fe-based amorphous coatings with fully amorphous or predominantly amorphous phases have been considered more promising for wear resistance applications. Guo et al. [22] reported the preparation of an almost completely amorphous Fe-based coating using arc spraying. The coating exhibited a dense structure with only 2% porosity and was predominantly composed of an amorphous phase. Similarly, Yao et al. [23] investigated FeCrNbBSiC amorphous coatings prepared by arc spraying, which displayed a porosity of 3.4% and contained both amorphous and a minor amount of nanocrystalline phases. In comparison with conventional Fe-based crystalline coatings, the Fe-based amorphous/nanocrystalline coatings exhibited significantly enhanced corrosion resistance and mechanical properties, attributed to their disordered structure and the strengthening effects of the dispersed nanocrystals [24,25].

Recent studies have shown that the incorporation of nanocrystals can significantly improve the wear resistance of Fe-based coatings [26]. For instance, Meghwal et al. [27] compared the microstructure, mechanical properties, and tribological behavior of fully amorphous and amorphous/crystalline Fe-based coatings. Their detailed analysis of the worn surfaces revealed that the amorphous/crystalline coatings experienced abrasive wear, surface fatigue, and minor oxidation at both room temperature and 600 °C. The enhanced hardness and wear resistance of these coatings were attributed to the presence of nanocrystals. Additionally, it has been reported that nanocrystals, generated by post-processing methods such as laser remelting, significantly improve the wear resistance of Fe-based amorphous coatings [28]. Jiang et al. [29] further demonstrated that laser remelting technology enhances the wear resistance of the coatings by precipitating hard phases or nanocrystals, which not only increases hardness but also changes the wear mechanisms from delamination, splitting, and plowing to a more stable wear and plowing process. However, up to now, there is limited research on the effect of nanocrystalline/amorphous phase content on the mechanical properties and wear resistance of Fe-based amorphous coatings, particularly those prepared by HVAS.

Although heat treatments such as annealing can enhance the hardness of Fe-based amorphous coatings by more than 12.5% and reduce the wear rates by approximately 30% [30], this method is somewhat complex. An alternative, more efficient approach is to optimize coating quality by adjusting process parameters, which is considered both economical and effective [31]. For instance, the quality of the coating can be improved without additional costs by fine-tuning parameters such as spraying voltage, current, distance, and air pressure. Tian et al. [32] prepared FeNiCrAl coatings via HVAS and investigated the influence of spraying voltage, current, and distance on hardness, porosity, and adhesive strength. They achieved a coating with the highest microhardness, adhesive strength, and minimal porosity through process optimization. Similarly, Luo et al. [33] studied the effect of spraying power on the wear resistance of Fe-based amorphous coatings using plasma spraying. They found that as the power increased from 30 kW and 35 kW to 40 kW, the porosity decreased from 7.96% and 6.13% to 5.75%, while the amorphous content of the coatings decreased from 96.07% and 73.89% to 65.54%, respectively. This indicates that both porosity and amorphous content gradually decrease with the increasing spraying power, which in turn leads to a reduction in wear loss. Unfortunately, most existing studies have focused primarily on optimizing basic coating indices by adjusting process parameters, without considering the actual service requirements [34].

In our previous work [35], the wear resistance of arc-sprayed FeCrMoWBRE amorphous/nanocrystalline coatings under higher loads, specifically for engine parts such as piston rings, cylinder liners, and crankshafts in heavy-duty vehicles, under oil and oil + sand lubrication conditions was investigated. It was found that abrasive and fatigue wear were the dominant wear mechanisms under low and medium loads (50–100 N), while abrasive, fatigue, delamination, and friction-induced wear mechanisms prevailed under higher loads (e.g., 125 and 150 N) for the coating. This work extends our previous research by focusing on the role of nanocrystalline/amorphous phase content in the mechanical properties and wear resistance of Fe-based amorphous/nanocrystalline coatings. In this study, FeCrMoWBRE coatings were prepared by HVAS, rendering optimal wear resistance comprehensively considering the working conditions of heavy-duty engine components under oil lubrication, and the influence of spraying power, including spraying voltage, current, and distance on porosity, amorphous phase content, microhardness, adhesive strength, and wear rate of the coatings is studied. Second, the relationship between nanocrystalline phase content and the microhardness, adhesive strength, and wear rate of the coating under actual working conditions is analyzed. Finally, the wear mechanism is discussed based on the analysis of the surfaces of the worn coating specimens. Hopefully, the results will be helpful in guiding the design and development of Fe-based amorphous/nanocrystalline coatings by HVAS for applications such as heavy-duty engine components.

## 2. Materials and Methods

### 2.1. Materials and Coating Deposition

The spraying material used in this study was a self-made flux-cored wire [35]. The flux-cored wire used in this study consists of a 434 stainless steel sheath enclosing a core powder mixture containing Cr, Mo, W, B, and RE elements, ensuring high thermal stability and wear resistance. It has the following chemical composition: Cr (18–20 wt.%), Mo (8–10 wt.%), W (2–4 wt.%), B (2–3 wt.%), RE (0.5–0.7 wt.%), and Fe (balance), and the outer shell of the flux-cored wire was made from 434 stainless steel, as listed in Table 1, while commercial 45# steel was used as the substrate. Prior to spraying, the substrate surface was roughly ground using 150-mesh coarse sandpaper. It was then ultrasonically cleaned with anhydrous ethanol to remove grease and contaminants. Next, the substrate was sandblasted using a 20-mesh white corundum abrasive at a sandblasting pressure of 0.6 MPa, a speed of 200 mm/s, and a distance of 250 mm to create a coarse surface. After sandblasting, the substrate was subjected to multiple ultrasonic cleanings with anhydrous ethanol to eliminate any remaining sand particles. Finally, the substrate was dried in a vacuum oven at 130 °C for 2 h to prevent oxidation.

An 8835 MHU HVAS system, which incorporates secondary atomization to enhance jet velocity and improve coating quality, was used for coating deposition. The air pressure for both the first and second atomization stages was set at 0.7 MPa, with a gun scanning speed of 500 mm·s^−1^ and a total of 10 passes. In this study, the Taguchi orthogonal array method was employed as the experimental design approach. Three levels each for spraying voltage, current, and distance were selected to create a set of orthogonal tests. The process parameters are summarized in Table 2, where the current and voltage are integrated into power to represent the input energy.

### 2.2. Coating Characterization

The as-sprayed coating samples were sectioned, ground, and polished following standard metallographic preparation procedures for characterization. The cross-sectional samples were prepared by embedding in epoxy resin, followed by sequential grinding with 2000-grit SiC paper, and polished with 1.0 µm diamond suspension. A field-emission scanning electron microscope (FESEM, Nova Nano 650, FEI Company, Hillsboro, OR, USA) was used to examine the surface, cross-sectional, tensile fracture, and worn surfaces of the samples, coupled with an energy dispersive spectrometer (EDS, EX250, EDAX Inc., Mahwah, NJ, USA) for compositional analysis. At a magnification of 1000×, fifteen random sections of each coating were selected for imaging. The porosity was then calculated using image processing software (Image J), with the average value reported [36]. Furthermore, high-resolution transmission electron microscopy (HRTEM, Tecnai G2 F20, FEI Company, Hillsboro, OR, USA) equipped with EDS was employed to observe the microstructure of the coatings and analyze the composition of localized regions. The phase structure of the coatings was analyzed by X-ray diffraction (XRD, AXS D8, Bruker Corporation, Billerica, MA, USA) with a Cu target (Kα radiation) and Ni filter (Kβ radiation). The operating parameters included an acceleration current of 100 mA, a voltage of 40 kV, a scanning range of 10–90°, a diffraction speed of 1.6°/min, and a step size of 0.02°. Phase identification was performed using HighScore Plus software (v3.0.5), and grain size calculations were carried out using Jade 6.5 software. The diffraction results were analyzed using the diffraction intensity comparison method, and the amorphous content was calculated by determining the ratio of the integral intensity of the amorphous diffuse peak region to the total integral intensity of the coating [37]. The thermal stability of the as-sprayed coatings was evaluated by differential scanning calorimetry (DSC, Pegasus DSC 404F3, NETZSCH, Selb, Germany), with the sample heated from room temperature to 1173 K under Ar gas protection at a heating rate of 10 K/min.

### 2.3. Mechanical Properties and Sliding Wear Behavior

The microhardness of the coatings was measured on the polished cross-section following the GB/T 4340.1-2009 standard, using a VTD402 micro-Vickers hardness tester (Taiming Instrument Co., Ltd., Beijing, China) with a 0.2 kg load and a 15 s holding time. Ten measurement points were selected at equal intervals from the coating/substrate interface, and the average value was reported as microhardness. The adhesive strength of the coatings was evaluated using a WE-10A universal material testing machine, according to the GB/T 9286-2020 standard (pull-off method), with a tensile rate of 0.8 mm/min. The measured value, obtained when the fracture occurred at the coating/substrate interface or within the coating itself, represented the adhesive strength of each coating.

The friction and wear tests of the coatings were performed using the MFT-5000 multifunctional friction and wear tester (RTEC Inc., Edison, NJ, USA) in accordance with the G133-05 standard. Prior to testing, each coating was wet-ground with 2000-grit SiC sandpaper, then finely polished and ultrasonically cleaned with anhydrous ethanol to ensure consistency in the experimental conditions. The friction pair consisted of a GCr15 steel ball with a diameter of 4 mm, while the specimen dimensions were 20 mm × 20 mm × 10 mm. A ball-on-disk wear test was conducted under oil lubrication at room temperature with a normal load of 100 N, a stroke length of 10 mm, a frequency of 5 Hz, and a duration of 30 min. The friction coefficient values were recorded and automatically reported by the tester. A 3D optical microscope (Contour Elite I, Bruker Corporation, Billerica, MA, USA) was used to capture the 3D morphology of the wear tracks and calculate the wear rate (R), which was determined using the following formula:

(1) $$R=\frac{S\cdot P}{F\cdot L},$$

where S (mm^2^) is the average cross-sectional area of the wear track, P (mm) is the amplitude, F (N) is the normal load, and L (m) is the total sliding distance. Each wear test was repeated at least three times to ensure data repeatability.

## 3. Results

### 3.1. Influence of Process Parameters on the Microstructure of the As-Sprayed FeCrMoWBRE Coating

#### 3.1.1. Surface and Cross-Sectional Characterization

**Figure 1:**
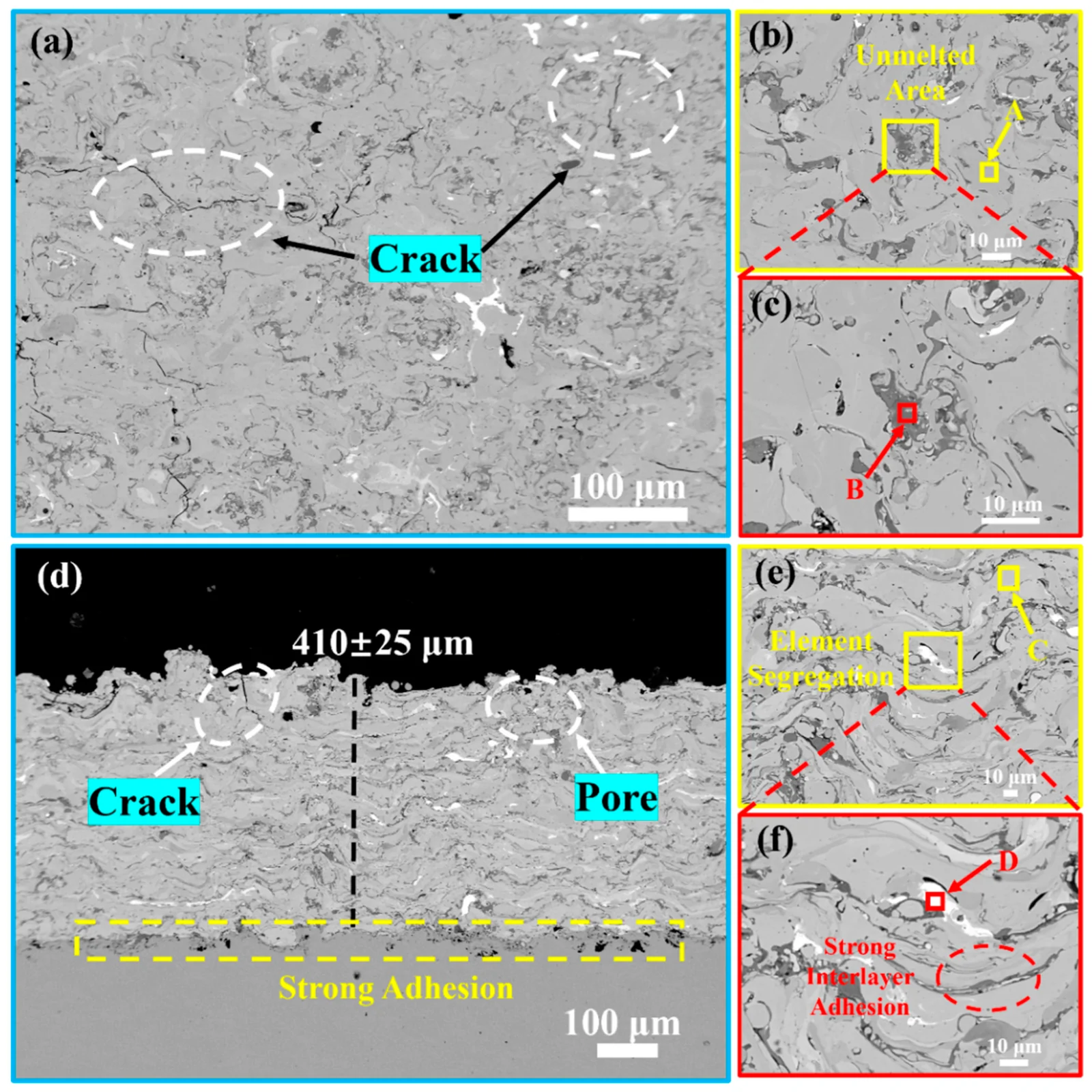
SEM images of a FeCrMoWBRE coating on the surface (**a**) and cross-section (**d**), as well as their magnified views (**b**,**c**,**e**,**f**), as an example.

The as-sprayed FeCrMoWBRE coating exhibits typical HVAS characteristics in both surface and cross-section. A sample deposited under medium spraying conditions (4.48 kW power, 180 mm distance) is selected to illustrate its detailed microstructure (Figure 1), representing a balance between coating density and mechanical properties. EDS analysis was conducted on regions with different grayscale levels: dark gray (A), black (B), white (C), and light gray (D), with the results summarized in Table 3. Figure 1a,d shows the surface and cross-sectional images of the coating, clearly revealing that the coating is dense, uniform, and exhibits a typical lamellar structure with a thickness of approximately 410 μm. Additionally, the interface between the coating and substrate is not distinct, indicating a good adhesion between the coating and the substrate. However, some small pores, cracks, and other defects are present in the coating. Figure 1b,c shows the presence of incompletely melted round particles and a small amount of oxides in the coating. Figure 1e,f further highlights that the interlayer adhesion is strong, with minor elemental segregation observed. The strong adhesion of the coating is attributed to the high-velocity air stream that accelerates the molten material, causing it to impact the substrate surface and form a dense interlayer structure. However, some small pores can form due to the volume shrinkage of the molten particles during solidification or the precipitation of gases from the molten material. These pores can serve as potential crack initiation sites, which will be discussed in detail in the following section. According to the EDS results, a small amount of W segregation, corresponding to the D region, is observed in the coating. This is due to the high melting point of W (approximately 3410 °C), which makes it difficult to completely melt during the spraying process, leading to elemental segregation. However, this W segregation can act as a reinforcing phase. When W particles accumulate on the substrate surface, they help prevent the agglomeration and uneven stacking of other particles, ensuring that the particles are evenly distributed on the coating surface. The uniform distribution of the particles reduces defects such as pores and cracks, thereby improving the coating’s density. As a result, the regions of W segregation do not form defects but rather contribute to a denser coating, which is reflected in both the surface and cross-sectional morphologies shown in Figure 1. Furthermore, the primary phase in the coating is phase C, with the mass fraction of W being approximately 8.02 wt.%. When W is sprayed together with other metal elements using arc spraying, its high melting point causes rapid cooling during the spraying process, leading to the formation of small, dense particles. Moreover, because W is less reactive with other elements, these particles help to limit the grain growth of other components in the coating. The presence of smaller grains results in a tighter structure, reducing the formation of microcracks and pores.

#### 3.1.2. Porosity of the FeCrMoWBRE Coatings

The FeCrMoWBRE coating, composed of individual deformed particles, exhibits a distinctive lamellar microstructure, commonly referred to as “splats”. However, these microstructural features inherently introduce pores, which can act as weak points in the as-sprayed coating, potentially compromising its wear and corrosion resistance [38,39]. To assess this, the porosity of the FeCrMoWBRE coatings was initially calculated using cross-sectional images.

**Figure 2:**
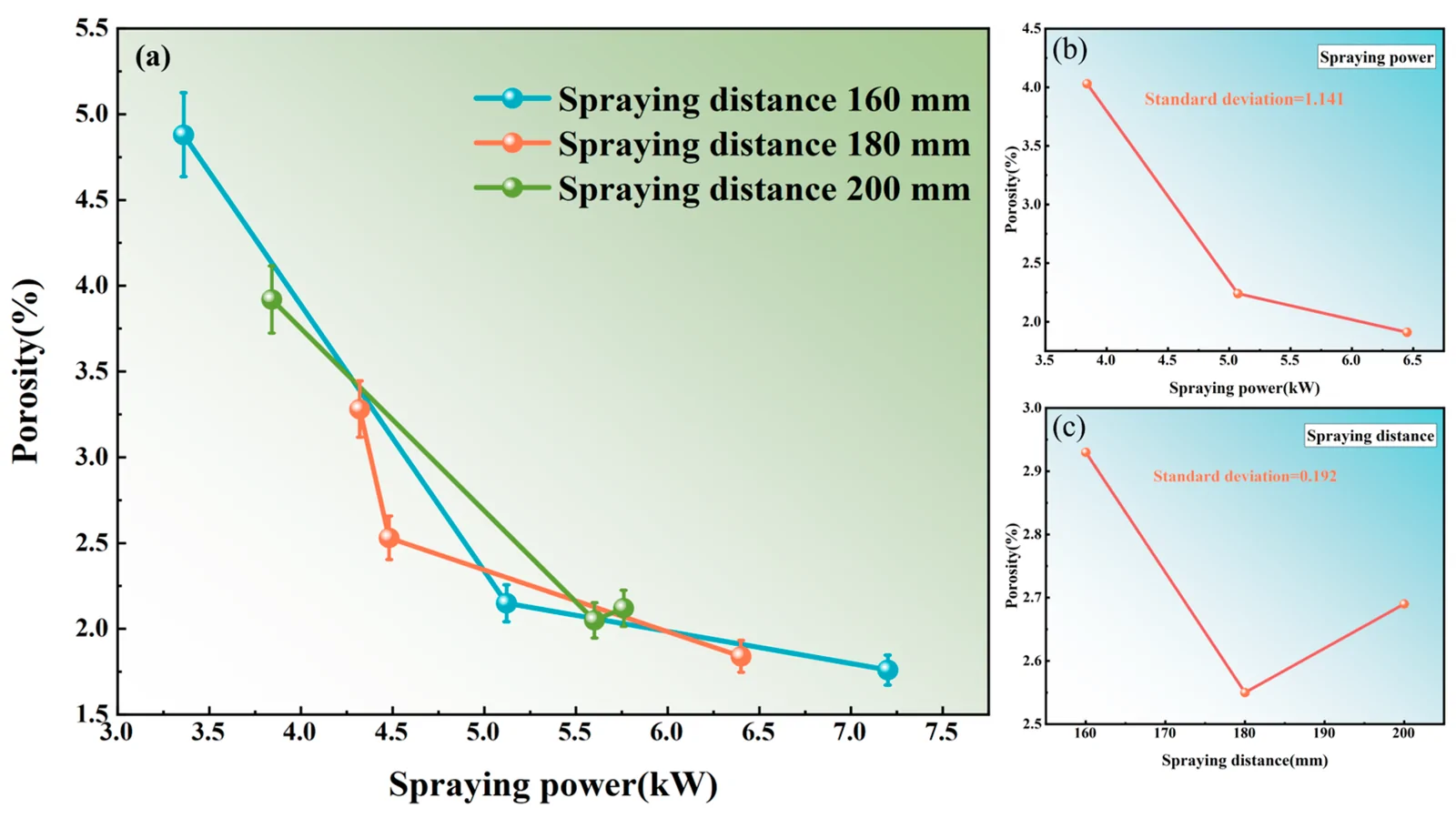
(**a**) The overall trend of the spraying power and the spraying distance on the porosity of the FeCrMoWBRE coating, and each effect of (**b**) spraying power and (**c**) spraying distance.

The porosity of the coatings, prepared under nine different process parameters, is presented in Figure 2. It is clear that porosity decreases with increasing the spraying power and the spraying distance. Among these two factors, spraying power exerts a more significant influence on porosity. This is evident not only from the trend observed in Figure 2b,c, but also from the experimental results, where the standard deviation of porosity is notably higher when varying the spraying power compared to changing the spraying distance. This is primarily due to the greater deformation resulting from the higher melting degree of the particles. Notably, when the spraying power exceeds 5 kW, the porosity stabilizes at approximately 2.0%.

**Figure 3:**
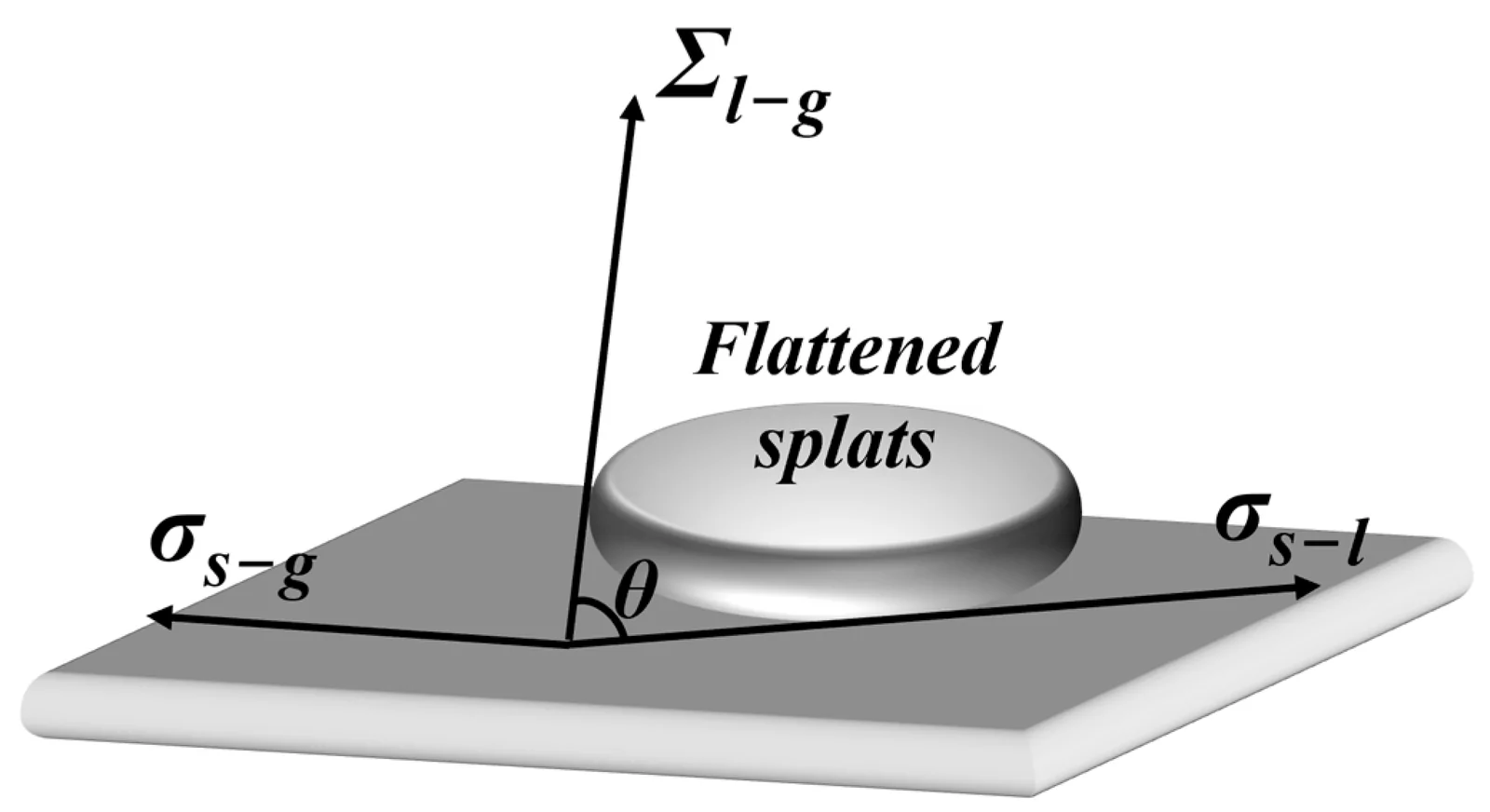
Schematic diagram of the wetting equation.

It is well established that higher spraying power corresponds to an increase in temperature in the deposition. As a result, even high melting point elements such as Mo and W are fully melted, reducing the surface tension of the molten material and thus leading to a decrease in porosity. The wetting equation can be expressed as follows:

(2) $$cos\theta =\frac{\sigma_{s-g}-\sigma_{s-l}}{\sigma_{l-g}},$$

where θ represents the contact angle, σ_s−g_ is the gas/solid contact surface tension, σs_−l_ is the solid/liquid contact surface tension, and σ_l−g_ is the liquid/gas contact surface tension, as depicted in the schematic diagram shown in Figure 3. As mentioned earlier, when the spraying power increases, the temperature also rises, enhancing the melting degree of the sprayed material. Consequently, σs_−g_ remains constant, while both σs_−l_ and σ_l−g_ decrease. This leads to an increase in cosθ and a decrease in the contact angle (θ), resulting in a better spreading of the droplets and, ultimately, a reduction in porosity [40]. It is assumed that when the spraying power exceeds 5 kW, the droplets are sufficiently melted, and further increases in the spraying power no longer significantly enhance the melting degree. As a result, the porosity of the as-sprayed coating stabilizes at a relatively constant value.

The spraying distance also affects the coating porosity by influencing the splashing behavior of droplets. With the secondary atomization spray gun pressure set at 0.7 MPa, the initial flight speed of the droplets is extremely high. Consequently, a shorter spraying distance leads to a higher droplet velocity, resulting in an increase in splattering. However, the spraying power has a more significant effect on the porosity of the HVAS coatings compared to the spraying distance. In this study, samples deposited at a spraying power of approximately 5 kW showed nearly identical porosity values (2.05–2.53%) regardless of the spraying distance. It is suggested that an optimal spraying distance can help reduce the velocity of molten particles upon reaching the substrate, thereby minimizing droplet splashing and coating porosity, while maintaining a balance with the spraying power within a certain threshold. These findings agree with previous research, which reported that higher particle velocities and higher temperature heat sources contribute to achieving a denser lamellar structure and reduced porosity in the Fe-based amorphous/nanocrystalline coatings [41].

**Figure 4:**
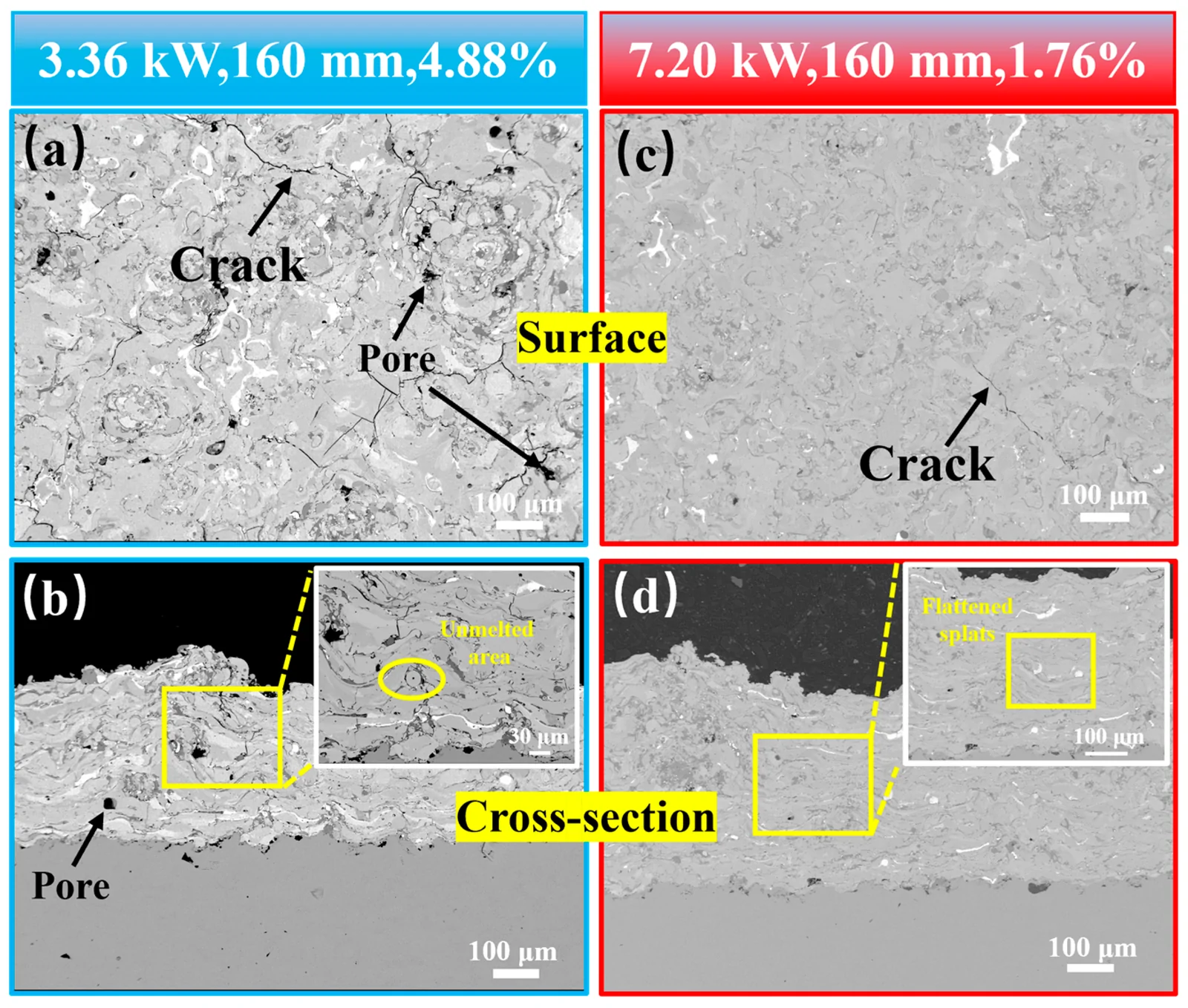
The surface and cross-sectional images of the FeCrMoWBRE coatings with the highest (**a**,**b**) and the lowest (**c**,**d**) porosity.

Figure 4 shows the surface and cross-sectional morphologies of the coatings with the maximum and minimum porosity in this study. A comparison of the two coatings reveals that the one with the highest porosity contains more unmelted particles and cracks. In contrast, the coating with the lowest porosity exhibits a well-defined laminated structure, with particles appearing flatter and more uniformly distributed, resulting in fewer defects such as holes and cracks. The microstructural analysis confirms that the process parameters significantly influence porosity in two ways: positively by enhancing the spreading degree of the sprayed particles on the substrate and negatively by increasing the splashing degree, which is closely related to the melting state and flight velocity of the sprayed particles. To control sintering-induced thickening and crack formation, careful optimization of process parameters, such as moderate spraying power and cooling rates, is essential. Additionally, post-deposition heat treatments at controlled temperatures can reduce internal stresses and enhance the coating’s stability in long-term service environments.

#### 3.1.3. XRD Pattern and Amorphous Phase Content (APC)

**Figure 5:**
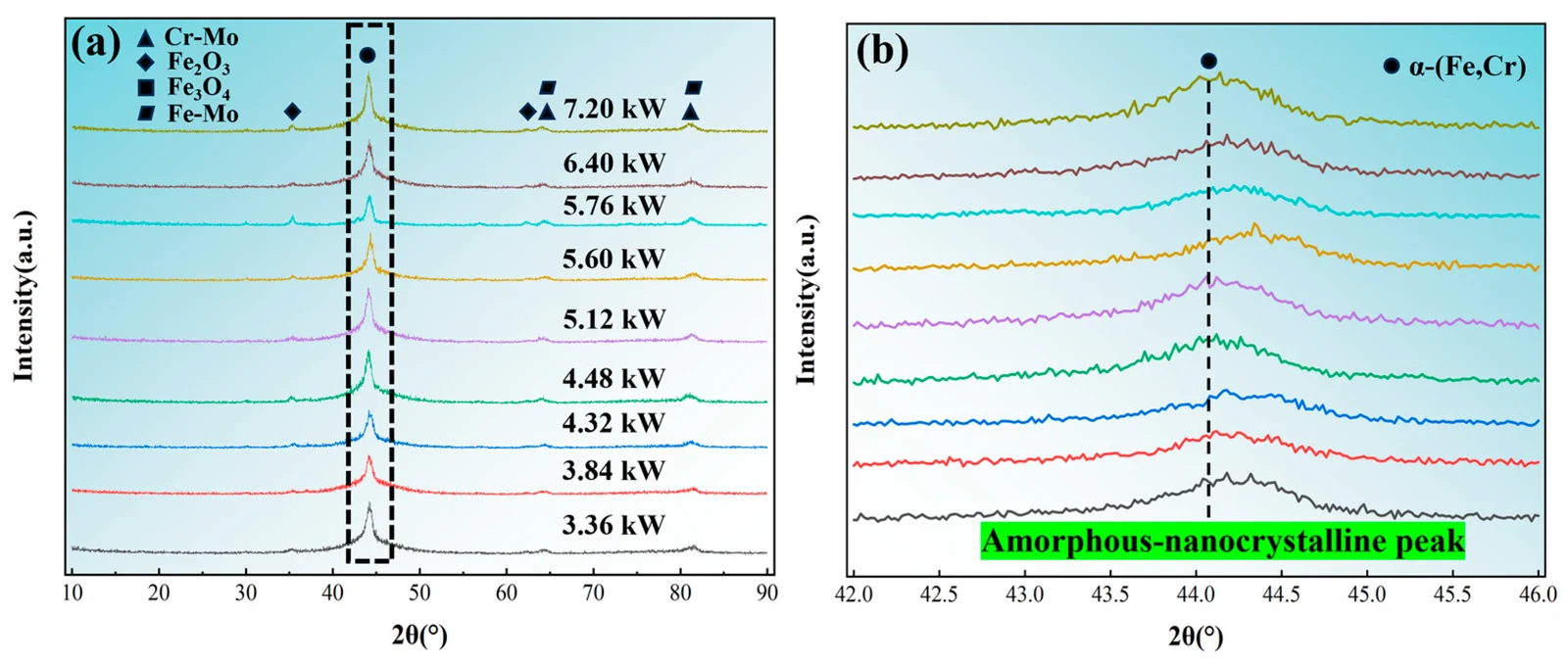
XRD patterns of the FeCrMoWBRE coatings under varying spraying powers and spraying (**a**) the enlarged view (**b**).

Figure 5 presents the XRD patterns of FeCrMoWBRE coatings fabricated under varying spraying powers and spraying distances, revealing a mixed amorphous-crystalline structure. The typical broad peak of the amorphous phase appears at 42~45°, accompanied by crystalline peaks, as shown in the magnified view in Figure 5b, which indicates that the as-sprayed FeCrMoWBRE coatings are primarily amorphous, with minor crystalline phases. The crystalline phases were identified as α-(Fe,Cr) (JCPDS 41-1466) as the dominant phase, with average grain sizes of approximately 22–29 nm, along with small amounts of Fe-Mo and Cr-Mo phases. Additionally, as shown in Figure 5b, a slight shift in the diffraction peaks toward higher 2θ angles compared to the standard PDF card suggests strain induced within the splats, likely due to the rapid cooling rates characteristic of the HVAS process. The additional peaks at 35.7° and 63° in the XRD pattern of the 5.76 kW sample correspond to Fe_2_O_3_, a secondary oxidation product (PDF No. 39-1346). This may have resulted from enhanced oxidation tendencies due to the higher spraying power. The APC was determined using the pseudo-Voigt function and MDI Jade software, as shown in Table 4. It is revealed that the APC in the FeCrMoWBRE coatings decreases progressively with increasing the spraying power and the spraying distance. For instance, the coating prepared at the lowest spraying power (3.36 kW) achieved an APC of 84.5%, whereas the coating prepared at the highest spraying power (7.20 kW) exhibited a reduced APC of 61.6%. These results indicate the sensitivity of the amorphous structure in FeCrMoWBRE coatings to the spraying parameters.

Moreover, the DSC thermograms for the FeCrMoWBRE coatings show a glass transition temperature (Tg) of approximately 713 K (440 °C) and an onset crystallization temperature (Tx) of around 811 K (538 °C). These results indicate the high thermal stability and excellent crystallization resistance of the coatings, as discussed in detail in our previous study [35].

The FeCrMoWBRE coating exhibits a relatively high APC, which decreases with increasing the spraying power due to the formation of a small amount of crystalline phases during deposition. As mentioned earlier, the high cooling rate inherent to the HVAS process facilitates the formation of the amorphous structure. However, at the same time, the elevated temperatures associated with arc spraying can disrupt the amorphous structure, leading to the nucleation and growth of crystalline phases. These crystalline phases likely originate from particles with high melting points that remain partially unmelted during spraying, subsequently serving as nucleation sites for crystallization through diffusion. The spraying power (arc power), P, directly influences the temperature and velocity of the sprayed particles and consequently affects the cooling rate and the substrate temperature during solidification [42]. Although the cooling rate during the HVAS coating solidification can reach magnitudes of 105 K/s [43], sufficient to meet the conditions for amorphous phase formation, an increase in spraying power elevates the in-flight particle temperature. This, in turn, reduces the cooling rate and extends the crystallization time, leading to a higher fraction of crystalline phases in the coating at elevated spraying powers. It is worth noting that higher particle temperatures and reduced cooling rates correlate with lower amorphous content in Fe-based amorphous coatings. The implications of this reduced amorphous content on mechanical properties and wear rate will be discussed in the following part.

Finally, it is important to highlight that the amorphous phase is thermodynamically metastable and naturally tends to transform into more stable phases, such as nanocrystalline or crystalline phases, in HVAS coatings. As a result, amorphous, nanocrystalline, and crystalline phases may coexist within the FeCrMoWBRE coatings [44]. Given the extremely high cooling rates associated with HVAS, the FeCrMoWBRE coating can be regarded as a composite material, consisting primarily of nanocrystalline particles embedded in an amorphous matrix, omitting the contribution of the crystalline phases. This interpretation is corroborated by the TEM analysis presented in the subsequent section.

#### 3.1.4. TEM Images

**Figure 6:**
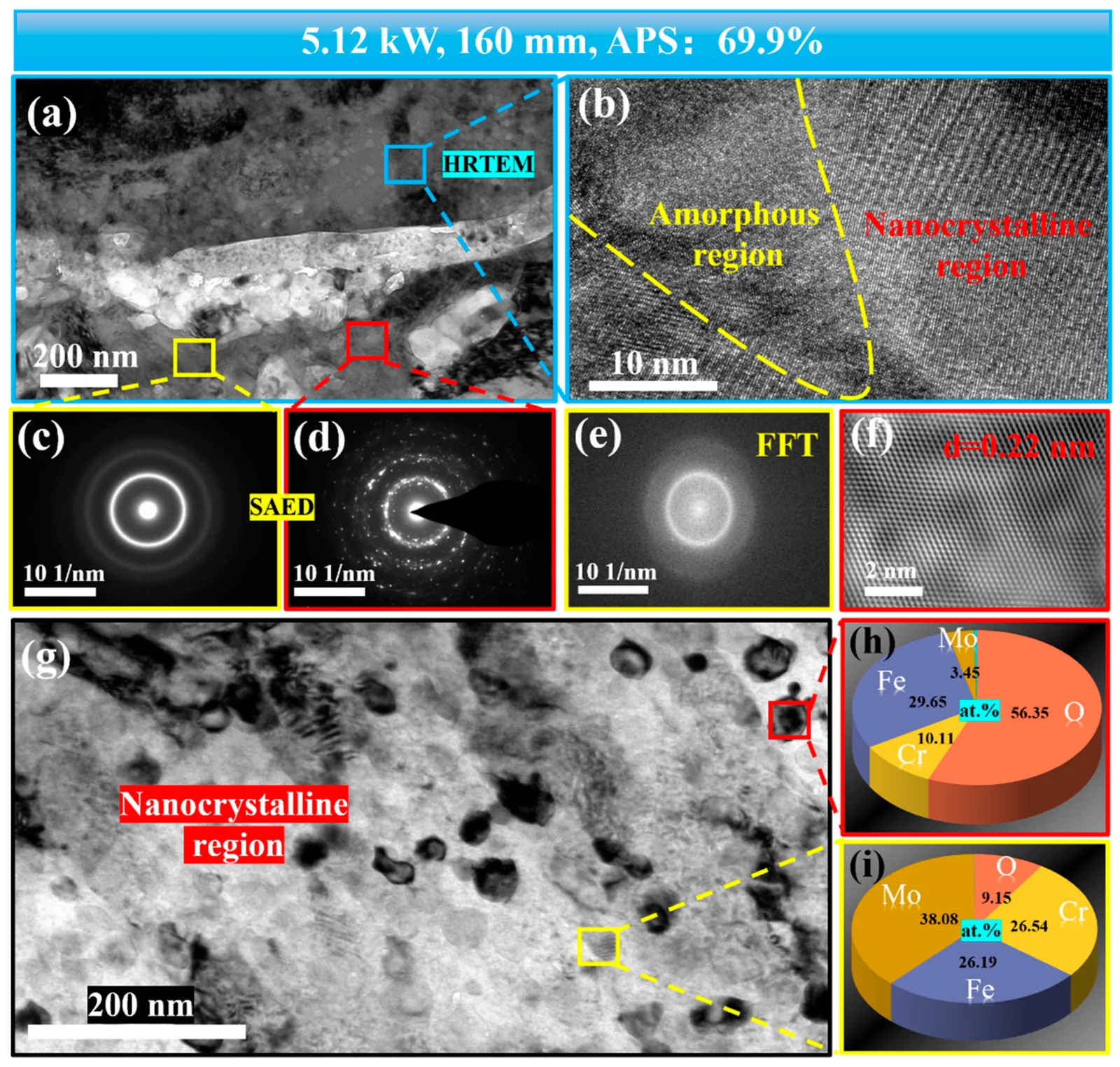
Microstructural information of the FeCrMoWBRE coating: (**a**,**b**) TEM and HRTEM images, (**c**–**f**) their corresponding SAED patterns (**c**,**d**), FFT results and magnified image of the nanocrystalline region (**f**), (**g**–**i**) TEM image of nanocrystals (**g**), and the EDS results (**h**,**i**).

The coexistence of the amorphous phase and nanocrystalline phase was further confirmed by the TEM results, as shown in Figure 6. Since no significant differences were observed among the samples prepared under varying spraying parameters, the FeCrMoWBRE coating fabricated with a spraying power of 5.12 kW and a spraying distance of 160 mm was selected as a representative example. This coating is supposed to have a relatively higher nanocrystalline phase content (30%).

The TEM and HRTEM images of the coating, along with their corresponding selected area electron diffraction (SAED) patterns, fast Fourier transform (FFT) results, and magnified views of the crystalline regions, are shown in Figure 6. The SAED patterns in Figure 6c,d, corresponding to the uniform and polycrystalline regions in Figure 6a, reveal diffuse diffraction rings with discrete bright spots, indicating a typical amorphous/nanocrystalline composite structure. Figure 6b provides an HRTEM image of the interface between the two phases in Figure 6a, revealing two distinct regions: one with a diffuse, long-range disordered structure typical of an amorphous phase, as confirmed by the diffuse rings in the FFT result (Figure 6e), and another with periodic lattice arrangements with an interplanar spacing of d = 0.22 nm, as shown in Figure 6f.

To further investigate the morphology and composition of the nanocrystalline phase in the coating, two representative regions from Figure 6g were analyzed using EDS, as shown in Figure 6h,i. The nanocrystals exhibit a typical spherical morphology with sizes ranging from 20 to 50 nm, which aligns closely with the grain size estimated from the XRD results (22–29 nm). The EDS analysis shows that the black nanocrystals contain approximately 56% oxygen, suggesting they are oxides. The light gray oxides are primarily composed of iron (Fe), chromium (Cr), and molybdenum (Mo). Combined with the XRD results, these nanocrystals can be identified as α-(Fe, Cr) solid solutions. Due to the high molybdenum content, approximately 38%, their oxygen content is relatively low. Molybdenum plays a significant role in enhancing the coating’s oxidation resistance and mechanical strength. Interestingly, both types of nanocrystals in the coating are oxidation products. While oxidation is typically undesirable and difficult to avoid in HVAS coatings, the oxidation-induced exothermic process in this coating facilitates the in situ formation of small, uniformly distributed nanocrystals, which positively contribute to the coating’s mechanical properties.

The formation of the amorphous phase is attributed to the multicomponent alloy system with high glass-forming ability and the extremely high cooling rates of the in-flight particles. In contrast, the formation of nanocrystalline grains is due to the crystallization of the original amorphous regions, which likely occurs in situ. This process may be related to preferential oxidation on the surface of in-flight particles and localized heating caused by thermal fluctuations during successive spraying [45,46]. It is worth emphasizing that the nanocrystals, with sizes ranging from 20 to 50 nm, are uniformly distributed within the amorphous matrix, indicating that the nanocrystalline grains may enhance the mechanical properties of the FeCrMoWBRE coating by reinforcing the amorphous matrix.

In summary, among the two critical factors affecting the quality of amorphous coatings, porosity and amorphous content [21], porosity can primarily be controlled by adjusting the spraying parameters, while the amorphous content in the amorphous/nanocrystalline FeCrMoWBRE coating can also be tailored through parameter optimization. Since both amorphous and nanocrystalline phases contribute to improving the hardness, the subsequent sections will discuss the specific effects of their respective contents on the mechanical properties of the amorphous/nanocrystalline FeCrMoWBRE coating.

### 3.2. Influence of Process Parameters on Microhardness and Adhesive Strength

**Figure 7:**
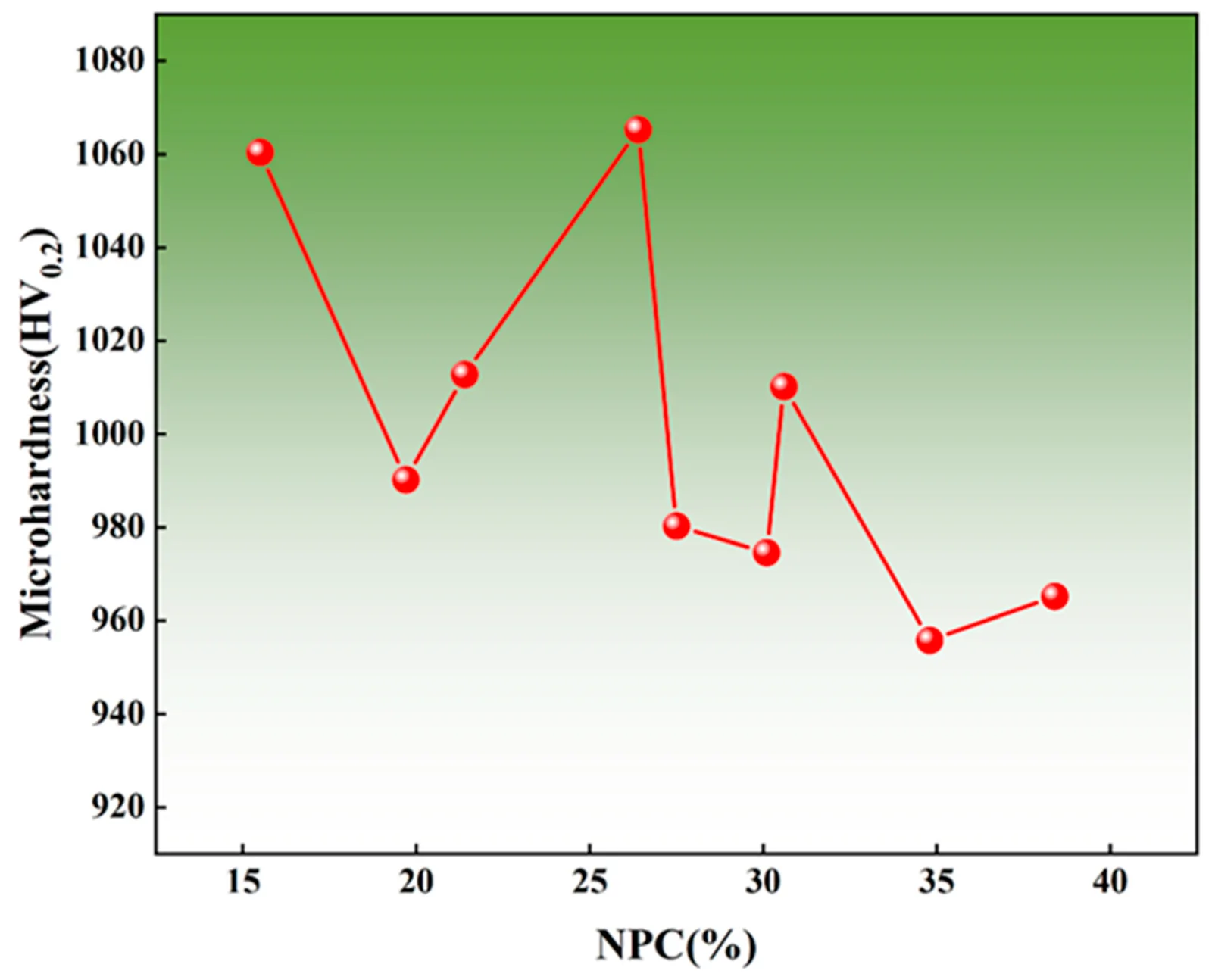
The influence of NPC on the microhardness of the FeCrMoWBRE amorphous/nanocrystalline coatings.

The microhardness values of the FeCrMoWBRE amorphous/nanocrystalline coatings prepared under nine different spraying parameters are presented in Table 5. It is evident that, as the spraying power increases, the microhardness of the coatings initially remains stable and then gradually decreases. In contrast, the microhardness decreases steadily as the spraying distance increases, although it seems a little confused between the spraying parameters and the microhardness, even not understandable that the microhardness decreases with the decreasing porosity until a relationship between nanocrystalline phase content (NPC) and microhardness is established, as shown in Figure 7. The results indicate that microhardness increases with the rising NPC, calculated as 100–APC, reaching a peak at a spraying power of 4.48 kW and a spraying distance of 180 mm, after which it declines with further increases in NPC. Considering that both porosity and NPC influence microhardness, the findings suggest that NPC plays a more dominant role in determining the microhardness of the FeCrMoWBRE amorphous/nanocrystalline coatings.

The microhardness of the FeCrMoWBRE amorphous/nanocrystalline coatings is primarily influenced by the incorporation of the amorphous phase as the dominant contributor, ultrafine grains (nanocrystalline phase) as reinforcements, and pores as attenuation factors. As previously noted, increasing the spraying power raises the temperature, allowing the spray materials to melt more completely, which leads to a reduction in porosity, as shown in Figure 2. Furthermore, the molten material possesses a significantly larger specific surface area compared to unmelted material of the same mass. Consequently, it becomes more susceptible to oxidation during the spraying process as the spraying power increases, which compromises the contribution of the amorphous phase to the coating’s microhardness. When the spraying power continues to rise, the specific surface area of the molten material no longer changes significantly. However, excessive heat leads to the crystallization of the amorphous phase in the coating material, resulting in the formation of additional nanocrystalline grains within the amorphous/nanocrystalline composite structure of the coatings [45]. It is widely recognized that fully amorphous coatings lack an ordered structure, making them more prone to elastic deformation under load. The presence of a crystalline structure, especially homogeneously distributed nanocrystalline grains, enhances the amorphous matrix by increasing its toughness. However, this enhancement diminishes and can even turn negative when the NPC becomes excessively high, disrupting the balance with the amorphous phase. Consequently, the microhardness of the FeCrMoWBRE amorphous/nanocrystalline coatings begins to decline when the spraying power exceeds 4.48 kW (corresponding to an NPC of 26.4%). Nevertheless, the rate of microhardness decrease becomes more gradual with further increases in NPC, which agrees perfectly with the observed relationship between the coating microhardness and the spraying power.

Spraying distance has the second impact on the microhardness of the FeCrMoWBRE amorphous/nanocrystalline coatings. As the spraying distance increases, the flight time of the spraying particles is prolonged, enhancing the exothermic oxidation effect of the molten spraying materials [46]. This leads to partial crystallization of the coating, which contributes to an increase in microhardness, as shown in Table 5.

The adhesive strength of the FeCrMoWBRE amorphous/nanocrystalline coatings, prepared under nine different process parameters, along with their variance results, is also summarized in Table 5. The results show that adhesive strength increases with rising spraying power, reaching a peak value of 60.75 MPa at a spraying power of 4.32 kW (with a similar value of 59.55 MPa observed at a neighboring spraying power of 4.48 kW). Beyond this point, the adhesive strength slightly decreases with further increases in spraying power and eventually shows a sharp decline at a spraying power of 7.2 kW (44.23 MPa). In the tested parameters, spraying power is identified as the most influential factor, followed by spraying distance. Higher spraying power enhances the melting of the spraying materials, leading to improved wettability and spreading on the substrate. Additionally, the high velocity of molten particles promotes the formation of a metallurgical bond both between the layers within the coating and at the coating/substrate interface, thereby increasing adhesive strength. However, excessive spraying power can cause an over-melting of the materials, resulting in a larger specific surface area of the molten particles. This, in turn, leads to more pronounced oxidation during the spraying process, ultimately degrading the adhesive strength [47].

It should be emphasized that, for all FeCrMoWBRE amorphous/nanocrystalline coatings, the fracture (deadhesion) occurred at the layer/layer surfaces within the coating rather than at the coating/substrate interface during the adhesive strength tests. This indicates that the adhesion strength between the coating and the substrate is higher than the layer/layer adhesion strength within the coating.

**Figure 8:**
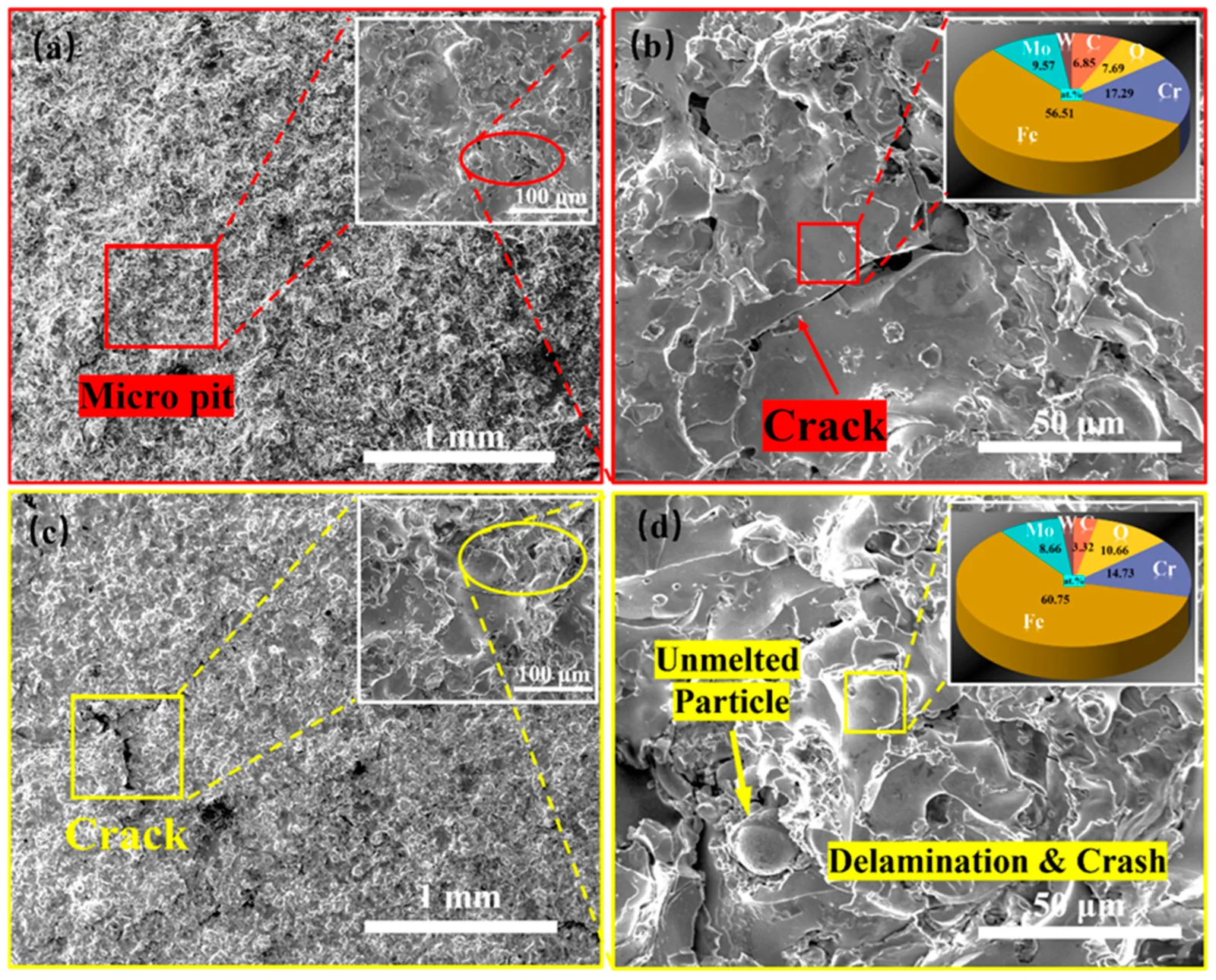
The fracture surface morphologies of the FeCrMoWBRE amorphous/nanocrystalline coatings with the highest (**a**,**b**) and the lowest (**c**,**d**) adhesive strength.

Figure 8 presents the fracture morphologies and EDS results of the coatings with the highest and lowest adhesive strength. As shown in Figure 8a,b, the fracture surface of the coating with the highest adhesive strength exhibits a flocculent structure with numerous micro-pits and a few localized microcracks. The EDS results at the microcrack regions indicate the presence of oxides, suggesting a fracture mechanism dominated by ductile fracture with some localized brittle fracture caused by oxides. In contrast, Figure 8c,d shows that the fracture surface of the coating with the lowest adhesive strength contains prominent macrocracks, a large number of delaminated structures, fragmented regions, and partially unmelted particles. The EDS results confirm that the delaminated areas are oxides, indicating a fracture mechanism primarily characterized by brittle fracture caused by unmelted particles and oxides. These differences suggest that the coating with higher adhesive strength exhibits stronger metallurgical bonding. Additionally, the fracture surface of the coating with lower adhesive strength contains more cracks, unmelted particles, and oxides, which agrees with the results mentioned above.

The spraying distance also plays a significant role in influencing the adhesive strength of the FeCrMoWBRE amorphous/nanocrystalline coatings. A shorter spraying distance allows the molten particles to reach the substrate more quickly, leading to improved mechanical bonding and higher adhesive strength. Conversely, when the spraying distance increases beyond a certain threshold, the velocity of the molten particles decreases substantially, making them more susceptible to oxidation.

In summary, the process parameters impact the adhesive strength of the FeCrMoWBRE amorphous/nanocrystalline coatings by altering the melting degree and velocity of the particles, thereby affecting the mechanical bonding between the molten particles at the layer/layer interface within the coating or the coating/substrate interface.

### 3.3. Influence of Process Parameters on Wear Resistance

Our objective is to develop an Fe-based coating using HVAS with optimized wear resistance, specifically designed for heavy-duty applications under oil lubrication conditions. The wear resistance of the FeCrMoWBRE amorphous/nanocrystalline coatings was assessed based on our previous study [35]. Additionally, considering the actual working conditions involve oil lubrication, this study primarily focused on coatings with a certain level of porosity to facilitate oil storage.

**Figure 9:**
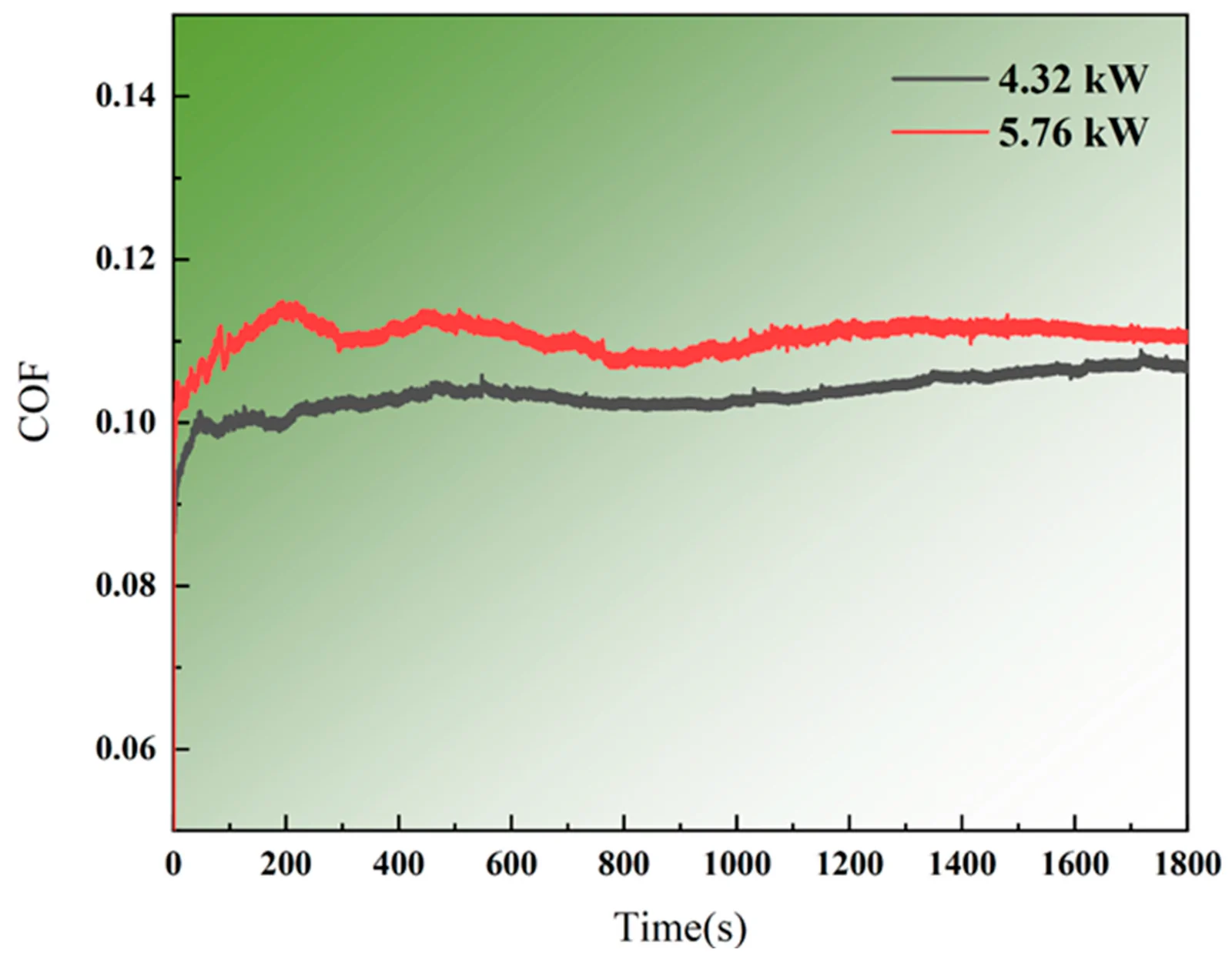
The coefficients of friction of the FeCrMoWBRE amorphous/nanocrystalline coatings deposited under spraying powers of 4.32 kW and 5.76 kW.

The coefficients of friction (COF) and wear rates of the FeCrMoWBRE amorphous/nanocrystalline coatings prepared under nine different process parameters are presented in Table 6. The results indicate that both the COF and the wear rate initially decrease with the increasing spraying power, reaching minimum values of 0.10 and 1.37 × 10^−7^ mm^3^·N^−1^·m^−1^ at a spraying power of 4.32 kW, respectively. However, these values increase slightly and then stabilize with further increases in spraying power. An exception is observed at a spraying power of 5.76 kW, where the COF and wear rate (0.11 and 1.73 × 10^−7^ mm^3^·N^−1^·m^−1^) are comparable to those at 4.32 kW. A detailed comparison of the two coatings with higher wear resistance reveals that, in terms of the COF, the value for the coating deposited at 4.32 kW remains more stable than that at 5.76 kW, as shown in Figure 9. Additionally, the wear rate of the former is 23% lower than the latter. Both advantages can be attributed to the optimal nanocrystalline phase content in the coating with the highest wear resistance.

The wear mechanism of the FeCrMoWBRE amorphous/nanocrystalline coating under both oil and oil + sand lubrication conditions has been thoroughly discussed in our previous work [35]. Therefore, this study focuses on explaining the specific results obtained herein. Generally, the wear resistance of a thermal-sprayed coating is primarily influenced by its microhardness, porosity, and adhesive strength. Theoretically, the optimal wear resistance for FeCrMoWBRE amorphous/nanocrystalline coatings would be achieved with maximum microhardness, minimum porosity, and maximum adhesive strength simultaneously. However, none of the coatings in this work meet all of these criteria, as shown in Figure 2 and Table 5.

Similarly to the microhardness discussed earlier, the wear resistance of the FeCrMoWBRE amorphous/nanocrystalline coatings is primarily determined by the incorporation of the amorphous phase as the dominant factor, ultrafine grains (nanocrystalline phase) as reinforcements, and pores as auxiliary factors for retaining oil within the coatings. Interestingly, a higher NPC does not appear to improve the wear resistance of the FeCrMoWBRE amorphous/nanocrystalline coatings. This is attributed to the reduction in fracture toughness (due to a decrease in APC), which promotes subsurface crack propagation and leads to increased delamination of the sprayed layers [48]. It is proposed that FeCrMoWBRE amorphous/nanocrystalline coatings with an appropriate NPC offer higher fracture toughness, better deformation accommodation, and superior impact resistance during the wear process under oil lubrication. This comprehensive advantage, stemming from microhardness, porosity, and adhesive strength, is observed in the coating with a 21.4% nanocrystalline phase, resulting in the highest wear resistance among the coatings in this work. Furthermore, a lower NPC is also expected to enhance the corrosion resistance of the FeCrMoWBRE amorphous/nanocrystalline coating, as crystalline phases are generally associated with reduced corrosion resistance [49].

**Figure 10:**
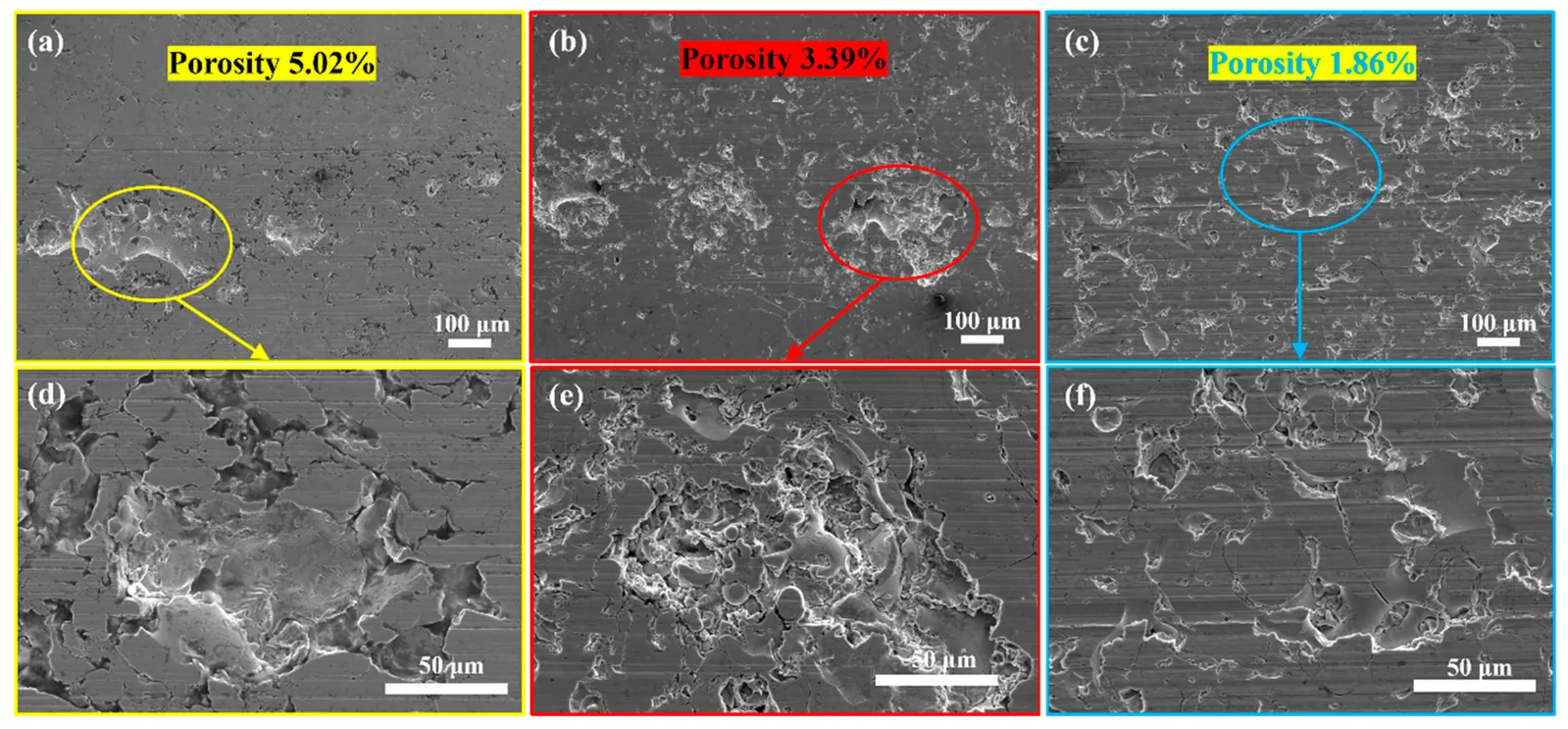
SEM micrographs of the worn surface of the FeCrMoWBRE amorphous/nanocrystalline coatings with the highest porosity 4.88% (**a**,**d**), medium porosity 3.28% (**b**,**e**), and the lowest porosity 1.76% (**c**,**f**).

It is worth noting that while pores in the FeCrMoWBRE amorphous/nanocrystalline coatings may enhance oil retention for lubrication, they are detrimental to wear resistance. To validate this, three coatings with the highest porosity (4.88%), medium porosity (3.28%, corresponding to the lowest wear rate), and lowest porosity (1.76%) were selected, and their wear tracks are compared in Figure 10. The coatings with the highest and medium porosity exhibit large areas of coating delamination, while the coating with the lowest porosity does not show such characteristics. This indicates that high porosity promotes pore expansion, causing extensive delamination under heavy loads and accelerating the wear process. Conversely, the coating with the lowest porosity shows significant wear characterized by numerous plastic grooves due to inadequate lubrication, resulting from insufficient oil retention. Considering both mechanical strength and oil lubrication, it is recommended that FeCrMoWBRE amorphous/nanocrystalline coatings with moderate porosity be employed for applications in oil-lubricated environments.

## 4. Conclusions

FeCrMoWBRE amorphous/nanocrystalline coatings with varying amorphous phase contents were successfully fabricated using HVAS technology, employing the Taguchi method with three factors and three levels. The effects of spraying power and spraying distance on the coatings’ microstructure (e.g., porosity, amorphous phase content), microhardness, adhesive strength, and wear rate under oil-lubricated conditions were systematically investigated. A clear correlation was established between the nanocrystalline phase content and the mechanical properties and wear resistance of the coatings. Furthermore, the roles of microhardness, adhesive strength, and porosity in influencing wear resistance were analyzed, taking into account practical application environments. These findings indicate that spraying power has a more significant impact on microhardness, adhesive strength, and wear resistance compared to spraying distance, primarily due to its influence on the nanocrystalline phase content. Achieving optimal wear resistance under oil-lubricated conditions requires a careful balance between microhardness, adhesive strength, and moderate porosity. In this study, the optimal coating featured an amorphous/nanocrystalline composite structure with a nanocrystalline phase content of 21.4%, porosity of 3.28%, microhardness of 1012.8 HV_0.2_, and adhesive strength of 60.75 MPa, resulting in the lowest wear rate of 1.37 × 10^−7^ mm^3^·N^−1^·m^−1^.

## Figures and Tables

**Table 1:** The chemical compositions of the cored wire material employed in this work.

Core Powder							Outer Sheath
Elements	Cr	Mo	W	B	RE	Fe	
Content (wt.%)	18–20	8–10	2–4	2–3	0.5–0.7	Balance	434 stainless steels

**Table 2:** Experimental conditions for orthogonal test design.

Factor No.	Arc Voltage (V)	Arc Current (A)	Spray Distance (mm)	Spraying Power (kW)
1	28	120	160	3.36
2	28	160	180	4.48
3	28	200	200	5.60
4	32	120	200	3.84
5	32	160	160	5.12
6	32	200	180	6.40
7	36	120	180	4.32
8	36	160	200	5.76
9	36	200	160	7.20

**Table 3:** EDS results of the regions labeled in Figure 1.

Elements (wt.%)	Fe	Cr	Mo	W	O
A	61.47	19.75	13.15	5.04	0.00
B	54.23	16.68	9.85	2.93	14.31
C	52.56	17.95	19.42	7.14	0.00
D	2.74	1.02	0.78	94.89	0.00

**Table 4:** Relationship between spraying power, distance, and the amorphous phase content (APC) of the FeCrMoWBRE coatings.

Spraying Power (kW)	Spraying Distance (mm)	APC (%)
3.36	160	84.5 ± 3.4
3.84	200	80.3 ± 3.1
4.32	180	78.6 ± 2.9
4.48	180	73.6 ± 2.8
5.12	160	69.9 ± 2.4
5.60	200	69.4 ± 2.4
5.76	200	72.5 ± 3.8
6.40	180	65.2 ± 2.3
7.20	160	61.6 ± 2.3

**Table 5:** Relationship between spraying power, distance, microhardness, adhesive strength, and nanocrystalline phase content (NPC) of the FeCrMoWBRE amorphous/nanocrystalline coatings.

Spraying Power (kW)	Spraying Distance (mm)	Microhardness (HV_0.2_)	Adhesive Strength (MPa)	NPC (%)
3.36	160	1060.4 ± 135.2	56.85 ± 7.2	15.5
3.84	200	990.2 ± 95.4	58.12 ± 6.8	19.7
4.32	180	1012.8 ± 122.1	60.75 ± 6.5	21.4
4.48	180	1065.3 ± 127.5	59.55 ± 5.9	26.4
5.12	160	980.3 ± 111.2	54.09 ± 6.3	30.1
5.60	200	920.4 ± 112.8	54.88 ± 6.2	30.6
5.76	200	1010.2 ± 125.7	54.13 ± 5.8	27.5
6.40	180	955.8 ± 88.9	53.57 ± 6.4	34.8
7.20	160	965.2 ± 83.5	44.23 ± 6.7	38.4

**Table 6:** Relationship between spraying power, distance, COF, and wear rate of the FeCrMoWBRE amorphous/nanocrystalline coatings.

Spraying Power (kW)	Spraying Distance (mm)	COF	Wear Rate (10^−7^ mm^3^·N^−1^·m^−1^)
3.36	160	0.14	3.18
3.84	200	0.12	2.21
4.32	180	0.10	1.37
4.48	180	0.12	2.76
5.12	160	0.13	2.56
5.60	200	0.12	2.44
5.76	200	0.11	1.73
6.40	180	0.13	2.58
7.20	160	0.12	2.40

## Data Availability

The original contributions presented in this study are included in the article. Further inquiries can be directed to the corresponding author.
